# Retraction: Tracheal Bronchus: A Rare Etiology of Recurrent Pneumonia in Children

**DOI:** 10.7759/cureus.r114

**Published:** 2024-01-25

**Authors:** Saif A Alqahtani, Anwar M Alghamdi, Rawan A Babader, Doaa A Aljehani, Razan K Alsultan, Rawiah Y Mushari, Maha Y Alyahya, Hawra I Alajwad, Ahmad A Alibrahim, Egbal B Altukruni, Reem N Alhasani, Arwa T Mandurah, Yara A Halabi, Batool J Alghamdi, Faisal Al-Hawaj

**Affiliations:** 1 College of Medicine, Jordan University of Science and Technology, Irbid, JOR; 2 College of Medicine, Ibn Sina National College for Medical Studies, Jeddah, SAU; 3 College of Medicine, AlMaarefa University, Ad Diriyah, SAU; 4 College of Medicine, Jazan University, Jazan, SAU; 5 College of Medicine, King Saud University, Riyadh, SAU; 6 College of Medicine, Arabian Gulf University, Manama, BHR; 7 College of Medicine, Medical University of Warsaw, Warsaw, POL; 8 College of Medicine, Qassim University, Qassim, SAU; 9 College of Medicine, Umm Al-Qura University, Mecca, SAU; 10 College of Medicine, Imam Abdulrahman Bin Faisal University, Dammam, SAU

The Editors-in-Chief have retracted this article. Concerns were raised regarding the identity of the authors on this article. Specifically, Faisal Alhaway and Malak Shammari have stated that they were added to this article without their knowledge or approval. The identity of the other authors could also not be verified. As the appropriate authorship of this work cannot be established, the Editors-in-Chief no longer have confidence in the results and conclusions of this article.

